# Prescription Medicines and the Risk of Road Traffic Crashes: A French Registry-Based Study

**DOI:** 10.1371/journal.pmed.1000366

**Published:** 2010-11-16

**Authors:** Ludivine Orriols, Bernard Delorme, Blandine Gadegbeku, Aurore Tricotel, Benjamin Contrand, Bernard Laumon, Louis-Rachid Salmi, Emmanuel Lagarde, on behalf of the CESIR research group

**Affiliations:** 1 Equipe Avenir prévention et prise en charge des traumatismes, Centre de recherche INSERM U897 “Epidémiologie et Biostatistiques,” Institut de Santé Publique d'Epidémiologie et de Développement (ISPED), Université Victor Segalen Bordeaux 2, France; 2 Service de l'évaluation, de la surveillance du risque et de l'information sur le médicament, Agence Française de Sécurité Sanitaire des Produits de Santé (Afssaps), Saint-Denis, France; 3 Université de Lyon, Lyon, France; 4 INRETS, Umrestte, UMR T 9405, Bron, France; 5 Service d'information médicale, CHU de Bordeaux, France; University of Liverpool, United Kingdom

## Abstract

**Background:**

In recent decades, increased attention has been focused on the impact of disabilities and medicinal drug use on road safety. The aim of our study was to investigate the association between prescription medicines and the risk of road traffic crashes, and estimate the attributable fraction.

**Methods and Findings:**

We extracted and matched data from three French nationwide databases: the national health care insurance database, police reports, and the national police database of injurious crashes. Drivers identified by their national health care number involved in an injurious crash in France, between July 2005 and May 2008, were included in the study. Medicines were grouped according to the four risk levels of the French classification system (from 0 [no risk] to 3 [high risk]). We included 72,685 drivers involved in injurious crashes. Users of level 2 (odds ratio [OR]  = 1.31 [1.24–1.40]) and level 3 (OR  = 1.25 [1.12–1.40]) prescription medicines were at higher risk of being responsible for a crash. The association remained after adjustment for the presence of a long-term chronic disease. The fraction of road traffic crashes attributable to levels 2 and 3 medications was 3.3% [2.7%–3.9%]. A within-person case-crossover analysis showed that drivers were more likely to be exposed to level 3 medications on the crash day than on a control day, 30 days earlier (OR  = 1.15 [1.05–1.27]).

**Conclusion:**

The use of prescription medicines is associated with a substantial number of road traffic crashes in France. In light of the results, warning messages appear to be relevant for level 2 and 3 medications and questionable for level 1 medications. A follow-up study is needed to evaluate the impact of the warning labeling system on road traffic crash prevention.

*Please see later in the article for the Editors' Summary*

## Abstract 2

Using three nationwide databases in France, Ludivine Orriols, Emmanuel Lagarde, and colleagues provide evidence that prescribed medicines contribute to the risk of experiencing a road traffic crash.

## Editors' Summary

About 1.3 million people die each year on the world's road. 90% of road traffic deaths occur in developing countries, with pedestrians, cyclists, and users of two-wheel vehicles (scooters, motorbikes) the most vulnerable road users. Although the use of prescribed benzodiazepines has already been documented to be associated with road traffic accidents in industrialized countries, the effects of other medicines have not been well studied or have inconsistent results (for example opioids and antidepressant medications). In the European Union, it is mandatory for pharmaceutical companies to provide data about the effect of a medicine on ability to drive. In France, a multidisciplinary group of experts was appointed to classify all medicines into four levels of risk (from level 0, no or negligible risk, to level 3, major risk), in terms of their effect on driving performances. In 2006, the International Council on Alcohol, Drugs and Traffic Safety proposed a classification list similar to the French classification system.

**Why Was This Study Done?** There is a pressing need to understand the association between prescribed medicines and the risk of road traffic crashes and also to have a more accurate picture of the fraction of road traffic crashes that are attributable to the use of prescribed medicines. This large French study aimed to advance knowledge in this important area.

**What Did the Researchers Do and Find?** The researchers used three data sources to find the information they needed: the national health care insurance database (which covers the whole French population and includes data on reimbursed prescription medicines), police reports, and the national police database of injurious road traffic crashes. Drivers involved in road traffic crashes (identified by their national healthcare number) between July 2005 and May 2008 were included in the study. The researchers used a statistical model to conduct a responsibility analysis, which determined factors associated with each driver responsible for the road traffic crash and each driver who was not responsible (controls). In addition, the researchers compared medicine exposure during a period immediately before the crash (case period) with exposure during an earlier period (control period) for each driver involved in a crash. The researchers retrieved data on reimbursed medicines, dispensed within six months of the road traffic crash, by linking included drivers to the national health care insurance database using their national ID, gender, and date of birth and grouped all prescribed medicines according to the four risk levels of the French classification system.

During the study period, 72,685 drivers involved in injurious road traffic crashes were included. The researchers found that drivers who had been prescribed level 2 and level 3 medicines were at higher risk (odds ratio 1.31 and OR 1.25, respectively) of being responsible for the road traffic crash, an association that remained after the researchers adjusted for the presence of chronic diseases. Furthermore, the researchers found that the fraction of road traffic crashes attributable to the use of (prescriptions for) level 2 and 3 medicines was 3.3% and that drivers were more likely to be exposed to level 3 medicines on the day of the road traffic crash than on a control day.

**What Do These Findings Mean?** This study provides strong evidence for the contribution of medicines to the risk of experiencing a road traffic crash. The French drug risk classification scheme seems accurate for medicines classified as levels 2 and 3 of risk for road traffic crashes. The effect on driving abilities of level 2 medicines depends both on the pharmacodynamics of the drug and on individual susceptibility, whereas for level 3 medicines, the pharmacodynamic effect seems to be predominant. The effects of level 1 medicines seem to be so dependent on individual susceptibility that effects on driving abilities are rare, which raises questions about the relevance of the labels for these medicines. However, some limitations with the study methodology might affect the interpretation of these findings. For example, the researchers used dispensing dates for medications as a surrogate for ingestion and were not able to check for noncompliance.

However, this study provides some of the strongest evidence to date of the need for health care workers to provide patients with proper information on the potential effect of any medicine that they are prescribed (or take) on their driving abilities.

**Additional Information** Please access these Web sites via the online version of this summary at http://dx.doi.org/10.1371/journal.pmed.1000366.

- The World Health Organization (WHO) provides information on road traffic accidents
- Two Web sites provide information for drivers about drugs that could affect their ability to drive
- The US National Institute on Drug Abuse of the National Institutes of Health has an information sheet on drugged driving

## Introduction

**Figure 1:**
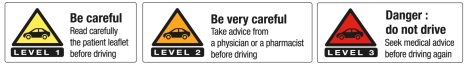
French medication labeling system.

The association between the use of benzodiazepines and the risk of road traffic crashes has now been documented with consistent results in several studies [1]–[13], but the effect of other medicines has been less assessed and results of available studies are often inconsistent [14]. This lack of assessment is particularly true for opioids [2],[8],[9],[12],[15],[16] and antidepressants [1],[12],[16],[17]. Psychoactive medicines may impair driving abilities owing to their action on the central nervous system (e.g., sedation in the morning following administration of a hypnotic), whereas other medicines may affect psychomotor functions by their action on physiological functions (e.g., hypoglycaemic seizures related to diabetic treatment) or because of centralised side effects (e.g., the depressant potential of carisoprodol on the central nervous system). In the European Union, it is mandatory for pharmaceutical companies to provide data about the effects of a medicine on the ability to drive and to use machinery prior to the medicine being allowed on the market. In 2003, the European Medicine Agency requested the standardized classification of medicines according to four levels of driving impairment risk, from level 0 (no or negligible risk) to level 3 (major risk), in order to provide health care professionals and patients with full information on the effects of medicines on driving abilities. The European DRUID project (Driving Under the Influence of Drugs, alcohol and medicines) identified 16 classification systems worldwide [18]. In 2006, the International Council on Alcohol, Drugs and Traffic Safety (ICADTS) proposed a medication classification system on the basis of the Belgium, Spanish, and French classification systems. In France, a multidisciplinary group of experts was appointed to classify all medicines according to four levels of risk in terms of their effect on driving performance [19]. A graded pictogram was designed to be printed on the outer packaging of all level 1 to 3 medicines (Figure 1). Pharmaceutical companies gradually implemented this policy from 2005 to 2008. Level 1, 2, and 3 medications are labeled with instructions that are relevant to driving for patients. The aim of our study was to estimate the association between medicine use, as estimated using prescribed medicine dispensation data from a health care reimbursement database, and the risk of injurious road traffic crashes, as well as the fraction of crashes attributable to medicine use in France.

## Methods

### Ethics Statement

This study was approved by the French Data Protection Authority.

### Data Sources

The study used three databases: the national health care insurance database, and two police databases referring to the same road traffic crash events but with different format and content.

#### Police reports

French police forces are required to fill out a police report for each injurious crash occurring in the country (about 70,000 reports each year). For some of the drivers involved in these injurious road traffic crashes, the national health care number (national ID) is recorded in the police report and can later be matched with medication dispensing records in the health care insurance database. Police reports are scanned and stored as image files. All available police reports in France were gathered over the study period.

#### National police database of injurious road traffic crashes

Police forces also collect details on each injurious crash event, which are stored in the national police database of injurious crashes (Bulletins d'Analyse d'Accident Corporel [BAAC]). This standardized database contains descriptive variables on the crash characteristics, the vehicles, and the people involved in the crash. Police forces also conduct additional investigations regarding injury severity from hospital records and categorize the people involved into four groups: unhurt, slightly injured, seriously injured (hospitalized more than 24 h), or killed (in the 30 d following the crash). All drivers involved in a road traffic crash are supposed to be tested for the presence of alcohol using a breath test. If this test is positive (≥0.5 g/l), the driver refuses to take the test, or the severity of the crash makes the test impossible, then the blood alcohol concentration is measured. If the breath test is negative, then the driver is registered as not being under the influence of alcohol. Missing data on alcohol impairment correspond to the following situations: the result of the blood measurement was unknown at the time of data entry in the database; the blood measurement could not be done (e.g., insufficient blood); the breath test was not done by the police; the breath test was positive but the blood alcohol concentration was not measured; or the breath test was negative but it was not coded in the database.

#### Health care insurance database

The national health care insurance database (Système National d'Informations Inter Régimes de l'Assurance Maladie [SNIIR-AM]) covers the entire French population (in 2008, 64,000,000 people) and includes data on reimbursed prescription medicines. A record is entered into the database each time a prescription medicine is dispensed to an outpatient at the pharmacy, including the national ID, the date dispensed, and the seven-digit code (CIP code) assigned to the medicine at the time of its marketing authorization. Data on long-term chronic diseases are also registered in this database, with the International Classification of Diseases, 10th edition (ICD-10) code), start, and end dates of the disease. In France, patients are fully reimbursed for health care expenses, including medicines, related to 30 recognized long-term chronic diseases [20].

### National ID Extraction and Matching Procedures

The first step of the study was extracting and matching data from the comprehensive French nationwide databases described above. Drivers involved in an injurious crash in France, between July 2005 and May 2008, were included through their national ID, gender, and date of birth, as extracted from police reports. An application, based on optical character recognition (OCR), was developed to automatically extract, from the image files, the crash date, an individual's national ID, gender, and date of birth. The extraction procedure was validated on a subsample of 293 police reports, which were printed and manually coded. A procedure was implemented to match each individual whose ID was extracted from police reports with the corresponding record from the national police database of injurious crashes. Two records were considered matched if six descriptive variables were in agreement. If a pair had three or more discordant variables, it was considered unmatched. For pairs with concordance for fewer than six variables and more than three variables, a probabilistic linkage method was developed [21]. When a decision could not be made automatically, pairs were reviewed by hand. Data on reimbursed medicines dispensed within 6 mo before the crash were obtained by linking included drivers to the national health care insurance database using their national ID, gender, and date of birth. Confidentiality was ensured by using the personal information anonymization function of the national health care insurance system [22].

### Medicines and Exposure Periods

Daily medication exposure was estimated for each pharmacotherapeutic class, according to the WHO Anatomical Therapeutic Chemical (ATC) classification. Medication exposure was calculated as starting one day after dispensing, and exposure duration was estimated from median values reported within a survey on medicine prescription in France [23]. This survey was conducted among 800 practitioners, representative of French physicians, three times a year, over a 7-d period. To ensure that prescribed medicines were not a consequence of the crash, medications dispensed on the crash day were not included in the analysis. We studied all dispensed and reimbursed prescription medicines grouped according to the French risk classification system [24].

A multidisciplinary group of experts developed the four-level risk classification system. The grading method analysed all available data: pharmacodynamic and kinetic effects, individual sensitivity, the conditions of use of each medicine, pharmacovigilance data, and experimental and crash study data [25]. This classification system ranks the four levels of driving impairment risk from level 0 (no or negligible risk) to level 3 (major risk). A graded pictogram is printed on the outer packaging of all level 1 to 3 medicines, accompanied by a written warning (Figure 1): level 0, medicines with no pharmacodynamic effect likely to alter the ability to drive, according to current information (6,282 medicines); level 1, medicines that do not generally impact on ability to drive, but require patient information (1,190 medicines); level 2, medicines that could affect the ability to drive and require medical advice before use (1,601 medicines); level 3, medicines that are known to affect the ability to drive during use (194 medicines).

### Determining Crash Responsibility

Responsibility levels in the crash were determined by a standardized method adapted from Robertson and Drummer [26]. This method, recently validated in France using the national police database of fatal crashes [27], takes into consideration the different factors likely to reduce driver responsibility: road, vehicle and driving conditions, type of accident, traffic rule obedience, and difficulty of the task involved. A score is assigned to each driver for each of these factors from 1 (favourable to driving) to 4 (not favourable to driving). The higher the sum of the scores, the more unfavourable the driving conditions, and thus the more likely it is that the driver will be considered not-responsible (nonresponsible) for the crash. Drivers were further grouped into two levels of crash responsibility: responsible (score <15) or nonresponsible (score ≥15).

This method of determining the driver's crash responsibility was approved by an independent expert responsibility evaluation (kappa  = 0.71).

### Analysis

#### Participant inclusion

Individuals whose police reports did not contain their national ID were not included. Drivers were censored at their first involvement in a road traffic crash in order to mitigate the impact of previous crashes on medicine exposure. We compared, by logistic regression, age, gender, injury severity, vehicle type, crash location, type of police force filing the police report, alcohol level, and responsibility status between included and nonincluded individuals.

#### Responsibility analysis

The purpose of the responsibility analysis is to compare exposure probabilities on the day of crash between responsible drivers (cases) and nonresponsible drivers (controls) [26]. This method ensures that both cases and controls are selected from the same driving population.

Statistical analyses were conducted using logistic regression. The associations between responsibility and age, gender, socioeconomic category, year, month, day of week, time of day, location, vehicle type, alcohol level, and injury severity were initially investigated using bivariate analysis; associated variables were included in the multivariate model when the *p*-value was less than 20% (Chi-squared test). This value was the case for all variables except for the year of crash, which was forced into the model because prescription patterns may have changed between the 2005–2006 and 2007–2008 periods. Further analyses adjusted for the presence of long-term chronic diseases. We tested the interactions between exposure and each of the adjustment variables.

Attributable fractions were estimated from the adjusted odds ratio (OR) estimates and the prevalence of exposure in responsible drivers [28]. Confidence intervals (CIs) were computed using the bootstrap method [29],[30], estimated from the 2.5th and the 97.5th percentiles of the distribution resulting from 500 simulations.

#### Case-crossover analysis

The case-crossover analysis consisted of a pair-matched analytical approach to compare medicine exposure during a period immediately before the crash (case period) with exposure during an earlier period (control period) for the same person [31]. We compared medicine exposure on the crash day with medicine exposure on the control day. The washout period between the case and control periods prevents any residual effect of an exposure in the control period on the case period. In France, the duration of a pharmacy-dispensed treatment cannot usually exceed 30 d (almost without exception, i.e., contraceptive pills), so the duration of the washout period was determined at 30 d. ORs were estimated by conditional logistic regression, using the PHREG procedure in SAS.

Data were analyzed using the SAS statistical software package, version 9.0 (SAS Institute Inc.).

## Results

### Study Population

The validation study conducted on 293 police reports showed that the national ID was recorded for 140 of the 455 drivers involved (28%). The automatic optical character recognition (OCR) software extracted 110 of these 140 national IDs (extraction rate  = 79%). Matching with the police national database of injurious crashes was possible for 90% of the IDs. The driver inclusion rate was thus expected to be about 20%.

**Figure 2:**
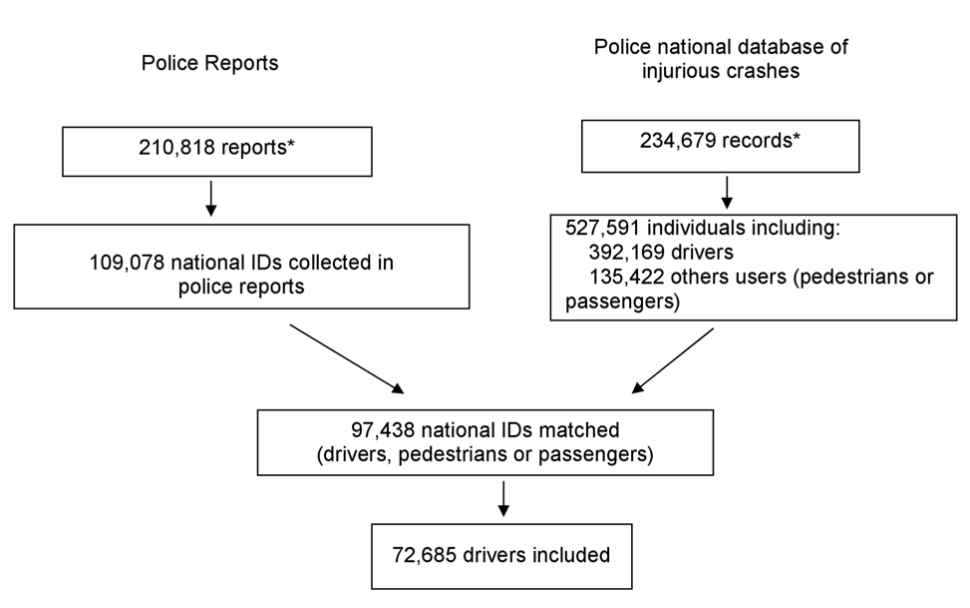
Flowchart of the inclusion procedure. *The discrepancy between the number of police reports and the number of records in the national police database of injurious crashes is explained by the fact that a small proportion of unavailable reports were being used for on-going further legal investigations.

Results of the overall extraction and matching procedures for the study are illustrated in Figure 2. We extracted 109,078 national IDs/gender/date of birth, from 210,818 police reports available from July 2005 to May 2008, corresponding to any individual involved in an injurious road traffic crash. Ninety percent of these individuals were matched with a corresponding record in the national police database of injurious crashes (72.8% fitted on all variables, 14.0% were matched by the probabilistic linkage method, and 3.1% manually). The linkage failed for 10% of the individuals, because the ID corresponded either to a driver involved in the crash but not captured in the national police database, or to an individual not involved in the crash (e.g., a witness, the owner of a vehicle involved).

This procedure led to the inclusion of 72,685 drivers (34,896 responsible and 37,789 nonresponsible drivers), i.e.,18.5% of the 392,169 drivers registered in the national police database of injurious crashes. Baseline characteristics of the study population are presented in Table 1. Injury severity was the main factor associated with the probability of being part of the study (OR  = 3.43 [3.29–3.58] for seriously injured drivers and OR  = 2.67 [2.57–2.77] for slightly injured drivers), thus explaining higher rates of inclusion for bicycle (OR  = 1.24 [1.16–1.33] and scooter drivers (OR  = 1.09 [1.03–1.16]) and drivers involved in nonurban accidents (OR  = 1.14 [1.10–1.18]), all of whom have been consistently documented in the literature to be more seriously injured. The inclusion rate was slightly lower for responsible drivers than for nonresponsible drivers (OR  = 0.91 [0.88–0.94]).

**Table 1:** Baseline characteristics.

Baseline Characteristics	*n*	Percent
**Total individuals**	72,685	
**Sex**		
Men	49,770	68.5
Women	22,915	31.5
**Age (y)**		
<18	3,055	4.2
18–24	14,814	20.4
35–34	16,666	22.9
35–44	15,488	21.3
45–54	11,796	16.2
55–64	5,990	8.2
65–74	2,837	3.9
≥75	2,039	2.8
**Socioeconomic category**		
Higher managerial and professional occupations	2,784	3.8
Intermediate occupations	24,984	34.4
Workers	11,887	16.4
Retired	6,449	8.9
Unemployed	3,021	4.2
Other/missing	16,014	22.0
Student	7,546	10.4
**Vehicle type**		
Light vehicle	42,792	58.9
Bicycle	3,867	5.3
Scooter	10,099	13.9
Motorbike	10,458	14.4
Commercial vehicle	2,550	3.5
Heavy goods vehicle	1,342	1.9
Other	1,577	2.2
**Injury severity**		
Unhurt	19,093	26.3
Slightly injured	26,327	36.2
Seriously injured	25,864	35.6
Killed	1,401	1.9
**Alcohol (g/l)**		
<0.5	58,700	93.5
[0.5–0.8]	568	0.9
[0.8–1.2]	786	1.3
[1.2–2]	1,392	2.2
≥2	1,320	2.1
**Long-term chronic disease**		
No	61,698	84.9
Yes	10,987	15.1

### Exposure to Medicines

Twenty seven percent (*n = *19,777) of the drivers included in the study were exposed to at least one prescribed medicine on the crash day. The proportion of drivers exposed to level 0, level 1, level 2, and level 3 medicines were respectively 21.6%, 10.2%, 11.4%, and 2.7%. There were 13,167 drivers (18%) exposed to at least one prescribed medicine of level 1, 2, or 3 (Table 2).

**Table 2:** Number of exposed drivers on the crash day by classification and number of medicines used.

*n* Medicines	*n* Exposed drivers
Level 0 medicines	15,715 (21.6%)^a^
* n* medicines of the level	
1	6,917
2	3,757
3	2,161
4	1,233
>4	1,647
No medicine in higher level^a^	6,610
Level 1 medicines	7,415 (10.2%)^b^
* n* medicines of the level	
1	5,681
2	1,361
3	315
4	49
>4	9
No medicine in higher level^a^	4,432
Level 2 medicines	8,268 (11.4%)^b^
*n* medicines of the level	
1	5,102
2	2,029
3	745
4	253
>4	139
No medicine in higher level^a^	6,753
Level 3 medicines	1,982 (2.7%)^b^
* n* medicines of the level	
1	1,724
2	234
3	23
4	1
No medicine in higher level^a^	1,982

a *n* drivers exposed to at least one medicine in the level and no medicine in any higher level.

b Exposed to at least one medicine of the risk level considered.

Table 3 shows the main pharmacotherapeutic drug classes used on the crash day among level 2 and 3 medicines by ATC class (third level of the ATC system).

**Table 3:** Level 2 and level 3 pharmacotherapeutic classes used on the crash day.

ATC Class	Level 2 Medicines	Level 3 Medicines
Total	13,147	2,265
**Alimentary tract and metabolism (A)**	1,056	—
*Insulins and analogues (A10A)*	370	—
*Blood glucose-lowering drugs, excluding insulins (A10B)*	668	—
**Cardiovascular system (C)**	196	—
*Antiadrenergic agents, centrally acting (C02A)*	195	—
**Musculo-skeletal system (M)**	277	—
*Muscle relaxants, centrally acting (M03B)*	248	—
**Nervous system (N)**	10,870	2,265
*Opioids (N02A)*	1,935	2
*Antimigraine preparations (N02C)*	337	—
*Antiepileptics (N03A)*	1,053	—
*Anti-Parkinson drugs (N04*)	175	—
*Antipsychotics (N05A)*	804	8
*Anxiolytics (N05B)*	2,843	471
Benzodiazepine derivatives (N05BA)	2,362	471
*Antidepressants (N06A)*	3,122	—
Selective serotonin reuptake inhibitors(N06AB)	2,188	—
*Hypnotics and sedatives (N05C)*	—	1,784
Benzodiazepine derivatives (N05CD)	—	295
Benzodiazepine-related drugs (N05CF)	—	1,196
Hypnotics and sedatives in combination, excluding barbiturates (N05CX)	—	293
*Drugs used in addictive diseases (N07B)*	443	—
Drugs used in alcohol dependence (N07BB)	69	—
Drugs used in opioid dependence (N07BC)	374	—
**Antihistamines for systemic use (R)**	327	—
*Phenothiazine derivatives (R06AD)*	216	—

Some drivers may have been exposed to several substances from the same pharmacological subgroup, explaining the difference with the number of exposed drivers presented in Table 2.

When adjusted for variables found to be associated with responsibility in the crash (age, gender, socioeconomic category, year, month, day of week, time of day, location, vehicle type, alcohol level, injury severity) and for medicines of others levels, the use of at least one level 2 or level 3 medicine was associated with the risk of being responsible for a crash (OR  = 1.31 [1.24–1.40] and OR  = 1.25 [1.12–1.40]). The use of level 0 medicines was associated with a decreased risk of being responsible for a crash (OR  = 0.92 [0.88–0.97]). The risk of being responsible was not significant for level 1 medicines (Table 4). The fraction of road traffic crashes attributable to use of levels 2 and 3 medicines was 3.0% [2.4%–3.5%] and 0.7% [0.4%–0.9%], respectively. The global fraction attributable to both level 2 and 3 medicines (considering exposure to level 2 or level 3 medicines on the crash day) was 3.3% [2.7%–3.9%]. The associations remained after adjustment for long-term chronic diseases (OR  = 0.92 [0.88–0.97] for level 0, OR  = 1.30 [1.22–1.38] for level 2, and OR  = 1.24 [1.11–1.39] for level 3). There was no interaction of medicine use with alcohol consumption (*p = *0.84 for level 2 and *p = *0.23 for level 3). The information on alcohol level was missing for 9,919 individuals (13.6%). Excluding these individuals from the univariate analysis led to no significant change in estimated ORs. We did not find any interaction between the use of level 2 or level 3 medicines and the adjustment variables.

**Table 4:** ORs for responsible road traffic crashes in users of prescribed medicines.

Medicine Level	Exposed Drivers	OR [95% CI]^a^	Exposed Drivers^b^	OR [95% CI]^c^	OR [95% CI]^d^
Level 0	15,715	0.92 [0.88–0.95]***	13,702	0.92 [0.88–0.97]*	0.92 [0.88–0.97]**
Level 1	7,415	0.96 [0.92–1.01]	6,478	0.96 [0.90–1.02]	0.95 [0.89–1.01]
Level 2	8,268	1.24 [1.19–1.30]***	7,102	1.31 [1.24–1.40]***	1.30 [1.22–1.38]***
Level 3	1,982	1.56 [1.42–1.71]***	1,679	1.25 [1.12–1.40]***	1.24 [1.11–1.39]**

Reference group, drivers not exposed to medicines of the risk level considered.

a Crude ORs.

b Model computed for the 62,766 drivers with no missing values for the adjustment variables.

c ORs adjusted for age, gender, socioeconomic category, year, month, day of week, time of day, location, vehicle type, alcohol level, injury severity and other level medicines.

d ORs adjusted for age, gender, socioeconomic category, year, month, day of week, time of day, location, vehicle type, alcohol level, injury severity, long-term chronic diseases, and other level medicines.

*p<0.01.

**p<0.001.

***p<0.0001.

Among level 2 medicines, the risk of being responsible for a crash was significantly higher for drugs used in diabetes (A10), antiepileptics (N03), psycholeptics (N05), psychoanaleptics (N06), and other nervous system drugs (N07). However, after Bonferroni correction for multiple testing, the association remained significant for the last four classes only (Table 5). The Benjamini and Yekutieli procedure based on the false discovery rate led to the same conclusions. The OR for level 3 psycholeptics was similar to the OR estimated for all level 3 medicines.

**Table 5:** ORs for responsible road traffic crashes in users of prescribed medicines by ATC class.

Level 2	Exposed Drivers^a^	OR [95% CI]^b^
Drugs used in diabetes (A10)	795	1.20 [1.03–1.40]*
Antihypertensives (C02)	172	1.07 [0.78–1.47]
Muscle relaxants (M03)	219	0.82 [0.62–1.09]
Analgesics (N02)^c^	1,845	1.04 [0.94–1.15]
Antiepileptics (N03)	755	1.41 [1.21–1.65]***
Anti-Parkinson drugs (N04)	125	1.15 [0.79–1.68]
Psycholeptics (N05)^d^	2,566	1.27 [1.15–1.40]***
Psychoanaleptics (N06)^e^	2,572	1.31 [1.19–1.44]***
Other nervous system drugs (N07)^f^	369	1.46 [1.16–1.84]**
Antihistamines for systemic use (R06)	267	1.05 [0.81–1.35]

a Model computed for the 62,766 drivers with no missing values for the adjustment variables.

b ORs adjusted for age, gender, socioeconomic category, year, month, day of week, time of day, location, vehicle type, alcohol level, injury severity, long-term chronic diseases, and other medicines.

c Including opioids (*n = *1,585), other analgesics and antipyretics (*n = *22), and antimigraine preparations (*n = *281).

d Including antipsychotics (*n = *558) and anxiolytics (*n = *2,250).

e Including antidepressants (*n = *2,509), psychostimulants (*n = *56), and antidementia drugs (*n = *33).

f Including drugs used in alcohol dependence (*n = *51), drugs used in opioid dependence (*n = *295), antivertigo preparations (*n = *7), and other nervous system drugs (*n = *16).

*p<0.05 (nonsignificant after Bonferroni correction α (corrected)  = 0.05/10 = 0.005).

**p<0.001.

***p<0.0001 (still significant after Bonferroni correction).

The risk of being responsible for a crash gradually increased from 1.14 [1.06–1.22] for users of one medicine of level 2 or 3 to 1.88 [1.58–2.25] for users of more than three medicines of level 2 or 3 (Table 6). Results from the case-crossover analysis showed a statistically significant association between the use of level 3 medicines and the risk of road traffic crash. There was no association with level 0, level 1, and level 2 medicines (Table 7).

**Table 6:** ORs for responsible road traffic crashes by number of level 2 and/or level 3 medicines used.

Number of Level 2/Level 3 Medicines	Exposed Drivers	OR [95% CI]^a^
0	55,264	Reference
1	4,259	1.14 [1.06–1.22]*
2	1,829	1.30 [1.17–1.43]**
3	817	1.86 [1.59–2.16]**
>3	597	1.88 [1.58–2.25]**

a ORs adjusted for age, gender, socioeconomic category, year, month, day of week, time of day, location, vehicle type, alcohol level, and injury severity.

*p<0.001 (still significant after Bonferroni correction).

**p<0.0001.

**Table 7:** Case-crossover analysis: ORs for road traffic crashes in users of prescribed medicines.

Medicine	Exposed Drivers^a^	OR [95% CI]^b^
Level 0	4,047	1.02 [0.98–1.07]
Level 1	2,249	1.02 [0.96–1.08]
Level 2	3,131	1.00 [0.95–1.05]
Level 3	896	1.15 [1.05–1.27]*

a Drivers exposed in the case period and not exposed in the control period.

b Only considering exposure to medicine of the highest level of risk.

*p<0.01.

## Discussion

We found evidence for an increased risk of being responsible for a road traffic crash for users of prescribed medicines defined as presenting a level 2 or level 3 risk of driving impairment according to the French medication classification system. The fraction of road traffic crashes attributable to levels 2 and 3 medicine use was 3.3% [2.7%–3.9%]. 

The study protocol planned for the inclusion of a large range of descriptive variables related to the crash and to the drivers involved. In particular, we were able to determine the responsibility status of the driver in the crash and to adjust for key confounding factors. The responsibility analysis is a real strength of the study as it allows for the comparisons of cases and controls that share the same characteristic of being drivers. In a previous study on the impact of illegal drug consumption, using the same national police database but limited to fatal crashes [27], the same method used to determine responsibility was approved by an independent expert evaluation of responsibility. Furthermore, because the responsibility analysis relies on the assumption that nonresponsible drivers are representative of the driving population, the authors of the previous study validated the comparison of a subset of the nonresponsible individuals with the driving population in France [27]. Finally, the strong dose-effect relationship found in our study between alcohol level and responsibility is a further indirect validation of the method. Importantly, responsibility levels were calculated independently of alcohol and illicit drug use because of their potential interactions with medicine use.

Medicine exposure was ascertained from computerized records of reimbursed prescriptions filled at the pharmacy. These data were not subject to underreporting, a major problem encountered when medicine exposure data is self-reported [5]. On the other hand, it is one of the study limitations that dispensing dates were considered in this study as a surrogate for actual consumption. We did not know whether the medicines were actually ingested or not. Noncompliance, which we were not able to check, would therefore result in exposure misclassification. Other studies using patient-derived data and the same dispensation database showed that the health care insurance data are reliable indicators of actual exposure for medicines used over a long time frame, less so for episodically used medicines [32]. We assumed that the exposure period started on the day after dispensing, as medicine dispensation on the day of crash may have been a consequence of the crash. Another limitation was that exposure to nonprescribed drugs can also not be estimated from the health care insurance database. However, less than 15% of medicines sold in France correspond to nonreimbursable medicines and most of these products have either no or negligible influence on the ability to drive.

The comparison between included drivers by means of their national ID and nonincluded drivers showed that injury severity was associated with the probability of being part of the study. Thus severely injured drivers were more likely to be included than slightly injured drivers. Killed drivers and uninjured drivers still had lower inclusion rates. This finding can be explained by the fact that injured drivers were more likely to be admitted to hospital so their health care number was more frequently noted in the police report. Thus, our study sample slightly overrepresented drivers injured in more severe crashes.

After adjustment for crash and individual variables, including exposure to other medicines, the risk of being responsible estimate was reduced for level 3 medicines, but the association did remain significant (from 1.56 [1.42–1.71] to 1.25 [1.12–1.40]). The crude risk of being responsible measured for level 3 medicines was thus partly related to these crash and individual variables and particularly due to a co-consumption of alcohol and level 2 medicines.

The protective effect of level 0 medicines could be explained by the treatment of those minor acute diseases that might lead to an increased risk of being responsible for the crash. Indeed, a number of specific physical and/or psychological conditions are likely to influence driving ability.

Surprisingly, we found no interaction between alcohol level, as reported by police forces, and medicine use, although alcohol is known to potentiate the effects of some medicines. It should be noted, however, that as the presence of alcohol is not always tested for in drivers involved in slight-injury crashes, this variable might be underestimated. Moreover, drivers who had a negative breath test were not tested for blood alcohol concentration (the legal limit in France is less than 0.5g/l). Information about illicit drug use was not available in any database. The analysis was also unable to adjust for driving exposure. Whilst on medication, some people may drive less to compensate for a perceived risk. They may also reduce their speed, pay more attention, or alter the road types that they use. The present study therefore estimated the impact of actual consumption and driving behaviors on the risk of road crash among active drivers.

According to our results, the French risk classification seems relevant regarding medicines classified as levels 2 and 3 of risk for road traffic crashes. Even if the risk for level 2 and 3 medications is similar, we believe that it is useful to differentiate these two levels. The effects of level 2 medicines on driving abilities depends both on the pharmacodynamics of the drug and on individual susceptibility; medical advice is therefore needed to weigh the potential risk for each individual. Various medicines are classified as level 2. The risks found for psycholeptics (mainly anxiolytics) and psychoanaleptics (mainly antidepressants) are concordant with others studies [2],[10]–[12],[16],[17]. The results for antiepileptics and other nervous system drugs (in particular medicines used to treat opioid dependence) are of interest and deserve further investigation. For some of the ATC classes in level 2, the association in the responsibility analysis was not significant; however, the number of drivers exposed to antihypertensives, muscle relaxants, anti-Parkinson drugs, and antihistamines for systemic use was small. On the other hand, despite a relatively large number of individuals exposed to analgesics (including opioid analgesics), we found no association with the risk of being responsible for a crash. With level 3 medicines, the pharmacodynamic effect is predominant so all users are advised not to drive. The effects of level 1 medicines may be so dependent on individual susceptibility that an effect on driving abilities might be a rare event. Therefore, the relevance of labeling level 1 medicines is questionable.

The respective roles of disease and the medicines used to treat disease are difficult to disentangle. After adjustment for the presence of a long-term chronic disease, results from the responsibility analysis did not suggest an important confounding effect of disease. In the case-crossover method, each individual is his or her own control and confounding due to individual factors is therefore eliminated, including fixed characteristics such as long-term chronic diseases. Other studies have used this approach to examine the relationship between medicines and the risk of injury [1],[12],[33]. The use of level 3 medicines was found to be associated with an increased risk of road traffic crash both in the responsibility analysis and in the case-crossover analysis. However, the risk associated with level 2 medicines in the responsibility analysis (OR  = 1.31 [1.24–1.40]) disappeared in the case-crossover analysis (OR  = 1.00 [0.95–1.05]). The risk of road traffic crashes associated with chronic exposure to level 2 medicines cannot be assessed by a case-crossover design. Indeed, an individual using a medicine throughout the study period would be exposed on the crash date and on the control day. Our results on level 2 medicines are therefore likely to be related to the impact of chronic medicine consumption, i.e., mainly drugs used in diabetes, opioids, antiepileptics, anxiolytics, and antidepressants. On the other hand, hypnotics and sedatives, mainly representing level 3 medicines, can be used on an acute basis and their impact on road traffic crashes are detected with the case-crossover analysis.

Our study provides evidence of the contribution of medicines to the risk of road traffic crashes. Improving driver behaviour is one of the challenges for improving road safety. Providing patients with proper information on the potential effect of medicines on their ability to drive is the main objective of drug and risk classifications such as the French framework. The European Union is currently aiming to harmonise drug classification systems, using a reliable methodology based on scientific evidence. This epidemiological study provides sound evidence for consideration in such an endeavour. A follow-up study is now needed to evaluate the effect of the French medication labeling system on the prevention of road traffic crashes.

## Abbreviations

ATC: Anatomical Therapeutic Chemical
CI: confidence interval
OR: odds ratio
